# Thrombocytopenia in Pregnancy: Clinical Challenges, Maternal–Fetal Risks, and Management Strategies

**DOI:** 10.3390/life16030462

**Published:** 2026-03-12

**Authors:** Sofoklis Stavros, Nikolaos Kathopoulis, Angeliki Gerede, Themos Grigoriadis, Efthalia Moustakli, Athanasios Zikopoulos, Nefeli Arkouli, Pavlos Machairoudias, Maria Tzeli, Ismini Anagnostaki, Dimos Sioutis, Konstantinos Louis, Anastasios Potiris

**Affiliations:** 1 Third Department of Obstetrics and Gynecology, University General Hospital “ATTIKON”, Medical School, National and Kapodistrian University of Athens, 12462 Athens, Greece; sfstavrou@med.uoa.gr (S.S.); dsioutis@gmail.com (D.S.); kostaslouisss@gmail.com (K.L.); 2 First Department of Obstetrics and Gynecology, Alexandra Hospital, Medical School, National and Kapodistrian University of Athens, 11528 Athens, Greece; nickatho@med.uoa.gr; 3 Unit of Maternal-Fetal-Medicine, Department of Obstetrics and Gynecology, Medical School, Democritus University of Thrace, 68100 Alexandroupolis, Greece; agerede@otenet.gr; 4 Department of Nursing, School of Health Sciences, University of Ioannina, 45500 Ioannina, Greece; ef.moustakli@uoi.gr; 5 Obstetrics and Gynecology, Royal Cornwall Hospital, Truro TR1 3LJ, UK; thanzik92@gmail.com; 6 Department of Obstetrics and Gynecology, Tzanio Hospital, 18536 Piraeus, Greece; narkoyli@windowslive.com; 7 Oxford University Hospitals NHS Foundation Trust, Oxford OX3 9DU, UK; 8 Department of Midwifery, Faculty of Health and Caring Sciences, University of West Attica, 12243 Athens, Greece; mtzeli@uniwa.gr; 9 Medical School, National and Kapodistrian University of Athens, 11528 Athens, Greece; isanagnostaki3@gmail.com

**Keywords:** autoimmunity, hypertensive disorders, HELLP syndrome, thrombotic microangiopathy, neuraxial anesthesia, peripartum hemorrhage, platelet transfusion

## Abstract

Thrombocytopenia affects up to 10% of pregnant women and represents the second most common blood disorder during pregnancy. Its causes include immune-mediated thrombocytopenia, hemolysis, elevated liver enzymes and low platelet count (HELLP) syndrome, preeclampsia (PE), and benign pregnant thrombocytopenia. Diagnosis is crucial because the cause dictates the effect on maternal health, pregnancy management, and neonatal outcomes. This narrative review examines the range of thrombocytopenia during pregnancy, primarily focusing on diagnostic evaluation, underlying pathophysiological causes, and differential diagnosis. In addition, it organizes maternal and fetal complications that might be caused by the condition, such as bleeding, preterm birth, and neonatal thrombocytopenia. Moreover, current patient management based on available evidence and clinical practice is discussed, including immunomodulatory therapies, platelet transfusions, clinical monitoring, and supportive care. A thorough and clinically guided approach to thrombocytopenia in pregnancy is indispensable for maximizing maternal and fetal outcomes and facilitating the personalization of perinatal care.

## 1. Introduction

Thrombocytopenia, defined as a platelet count of <150 × 10^9^/L, affects approximately 7–10% of pregnancies. Gestational thrombocytopenia accounts for about 70–80% of cases, while immune thrombocytopenia (ITP) occurs in approximately 3–5%, and pregnancy-related conditions such as PE and HELLP syndrome account for roughly 5–10% of cases [1,2,3]. Pregnancy-related changes, such as increased plasma volume and higher platelet consumption, may be responsible for mild reductions in platelet counts. However, because pathological thrombocytopenia may adversely affect the health of both the mother and the fetus, it requires careful assessment [3,4].

Gestational thrombocytopenia is the most common cause of thrombocytopenia in pregnancy and is a benign condition characterized by mild-to-moderate thrombocytopenia, with onset in the second half of pregnancy and spontaneous resolution after delivery [5,6]. However, the mother and fetus may be at serious risk from other reasons such as thrombotic microangiopathy, autoimmune thrombocytopenia, and hypertensive diseases of pregnancy (like PE and HELLP syndrome). These disorders may be associated with severe thrombocytopenia, bleeding complications, organ dysfunction, preterm labor, and increased perinatal morbidity and mortality [7,8,9].

Differentiating between the many causes of thrombocytopenia during pregnancy remains a diagnostic challenge since various causes have been linked to identical laboratory results and clinical characteristics [10]. The time of onset, severity of thrombocytopenia, maternal symptoms, and presence of other laboratory abnormalities are factors that help in making an accurate diagnosis. Early identification of high-risk characteristics is essential [5,11]. Therapeutic options differ substantially, and delays can result in serious adverse outcomes. Thrombocytopenia also affects the safe use of neuraxial anesthesia, decisions about the timing of intervention, and the choice of delivery mode [12,13]. Fetal and neonatal risks such as intrauterine growth restriction, preterm birth, neonatal thrombocytopenia, and cerebral hemorrhage are different according to the underlying cause. In this context, examples of maternal complications include pre- and postpartum hemorrhage, an increased need for transfusions, and limited anesthetic options [14].

Providing optimal care necessitates a multidisciplinary approach that includes obstetricians, hematologists, anesthesiologists, and neonatologists. This study provides a comprehensive overview of thrombocytopenia in pregnancy, covering its pathophysiology, diagnostic challenges, maternal and fetal risks, and treatment strategies. By integrating recent research and treatment recommendations, the article supports early diagnosis, individualized care, and optimal outcomes for both mother and child.

Despite advancements in hematological and obstetric care, diagnosing and treating thrombocytopenia remains challenging, particularly when clinical symptoms overlap or present atypically. The importance of evidence-based and clinically focused evaluations is underscored by recent treatment options and the continually updated guidelines on platelet thresholds for labor and anesthesia.

## 2. Methodology

The aim of this narrative review is to provide a comprehensive and clinically oriented overview of thrombocytopenia in pregnancy, with particular emphasis on the etiology, diagnostic challenges, maternal and fetal risks, and therapeutic strategies. To inform this narrative review, a structured literature search was conducted in PubMed, Scopus, and Web of Science databases. The search included combinations of the following keywords: “thrombocytopenia”, “pregnancy”, “gestational thrombocytopenia”, “immune thrombocytopenia”, “preeclampsia”, “HELLP syndrome”, and  “thrombotic microangiopathy”. Articles published in English between January 2000 and January 2026 were considered, with particular emphasis on studies published within the most recent five to seven years.

Studies were selected based on their clinical relevance to thrombocytopenia in pregnancy, including epidemiology, pathophysiology, diagnostic evaluation, maternal–fetal outcomes, and management strategies. As this study represents a narrative review rather than a systematic review, a formal quantitative synthesis or PRISMA-based screening process was not performed. Instead, the literature was qualitatively evaluated to provide a clinically oriented overview of the topic.

## 3. Physiological Changes in Platelet Count During Pregnancy

During normal pregnancy, dynamic hematological changes affect both platelet number and function. Increased platelet activation, disturbances in their synthesis and consumption, and expansion of plasma represent the main underlying mechanisms. Therefore, a mild reduction in platelet count is common during pregnancy and should be distinguished from pathological thrombocytopenia [15].

The progressive increase in plasma volume leads to hemodilution and a relative decrease in circulating platelet concentration, mainly during the second and third trimesters, without reflecting a real decrease in total platelet mass [16]. In addition, increased platelet consumption within the uteroplacental circulation, where they play a crucial role in maintaining the vascular integrity of the placenta, results in enhanced platelet turnover [17,18].

Pregnancy is also characterized by a prothrombotic state associated with increased platelet activation and aggregation. Platelet adhesion and consumption are encouraged by increased procoagulant factors and endothelial activity, which helps to reduce platelet count to a small extent [19,20]. Despite these physiological alterations, most pregnancies are able to produce sufficient platelets because bone marrow megakaryocyte activity often remains normal or slightly increased [21,22,23]. Physiological pregnancy-associated thrombocytopenia is typically mild, with platelet counts rarely falling below 100 × 10^9^/L, and is not associated with bleeding or adverse maternal or fetal outcomes [24]. It is worth noting that coagulation parameters are often within normal limits and platelet function is mostly preserved. Within a few weeks after delivery, platelet counts usually return to pre-pregnancy levels, indicating that these changes are benign and reversible [25].

Understanding the normal decrease in platelet count during pregnancy is essential to avoid unnecessary diagnostic procedures or therapeutic interventions. Differentiation between benign and pathological thrombocytopenia requires an integrated assessment of platelet trends, gestational age, clinical context, and associated laboratory findings. Recognition of these normal changes provides the foundation for accurate diagnosis and appropriate management of thrombocytopenia in pregnancy [26,27]. Recent studies indicate that pseudothrombocytopenia may account for a substantial proportion of cases initially classified as severe thrombocytopenia, representing up to one-fifth of cases in some series, underscoring the importance of confirming the diagnosis before initiating potentially unnecessary therapeutic interventions.

## 4. Etiology of Thrombocytopenia in Pregnancy

Pregnancy-related thrombocytopenia comprises a heterogeneous group of conditions with distinct clinical features, underlying pathophysiological mechanisms, and management strategies [5,28]. Since the etiological spectrum ranges from benign pregnancy-related disorders to serious pathological entities associated with increased maternal and fetal morbidity, accurate identification of the underlying cause is essential [29]. In contrast to gestational thrombocytopenia, which is typically benign and self-limited, conditions such as thrombotic microangiopathies and severe PE or HELLP syndrome may represent medical emergencies that can rapidly progress to multiorgan dysfunction and therefore require urgent multidisciplinary management.

The principal causes include gestational thrombocytopenia, ITP, PE, HELLP syndrome, thrombotic microangiopathies, and secondary etiologies [27]. Table 1 lists the major causes of thrombocytopenia during pregnancy along with their unique characteristics.

From a clinical perspective, gestational thrombocytopenia generally represents a benign condition requiring observation only, whereas ITP may require targeted therapy depending on platelet counts and bleeding symptoms. In contrast, conditions such as TTP, atypical hemolytic uremic syndrome, severe PE, and HELLP syndrome require urgent evaluation and prompt treatment due to the risk of rapid maternal and fetal deterioration.

### 4.1. Gestational Thrombocytopenia

About 70–80% of occurrences of thrombocytopenia during pregnancy are caused by gestational thrombocytopenia. Platelet counts typically remains above 100 × 10^9^/L, and it is characterized by mild thrombocytopenia. It typically appears in the late second or third trimester. Women with this condition typically have no symptoms and no prior history of thrombocytopenia outside of pregnancy [35,36].

Although the exact cause of gestational thrombocytopenia is unknown, it is believed to result from normal hemodilution, increased platelet clearance, and increased platelet consumption in the uteroplacental circulation [4,6]. It is crucial to remember that this condition is benign, unrelated to neonatal thrombocytopenia, and does not raise the risk of maternal bleeding or unfavorable fetal outcomes. Platelet counts usually return to normal within a few weeks after delivery, although recurrence in subsequent pregnancies is common, though typically mild [37].

### 4.2. ITP

Autoimmune thrombocytopenia is characterized by decreased platelet production and increased antibody-mediated platelet destruction. It accounts for approximately 3–5% of pregnancy-related thrombocytopenia and can be diagnosed either before or during pregnancy. In contrast to thrombocytopenia of pregnancy, its severity can range from mild to severe and often presents early in pregnancy [38,39].

Although many women remain asymptomatic, the severity of thrombocytopenia is associated with an increased risk of maternal bleeding [13]. Transplacental transfer of antiplatelet antibodies, which can cause neonatal thrombocytopenia, is a major concern in autoimmune thrombocytopenia. Close monitoring of the newborn’s platelet count is recommended, although severe bleeding is rare. Differential diagnosis from thrombocytopenia of pregnancy is critical due to differences in management [40,41].

### 4.3. Hypertensive Disorders of Pregnancy

Pregnancy-related conditions associated with thrombocytopenia include preeclampsia and HELLP syndrome, both of which may present with significant maternal morbidity. Thrombocytopenia in these conditions is mainly attributed to endothelial dysfunction, platelet activation, and increased microvascular platelet consumption [4,42].

Although the thrombocytopenia associated with PE may develop rapidly, it is usually mild to moderate in severity. In contrast, HELLP syndrome is characterized by severe thrombocytopenia and is associated with significant maternal morbidity, including acute kidney injury, disseminated intravascular coagulation, and hepatic parenchymal rupture [7,43,44]. Emergency delivery often remains the only definitive treatment option, and these disorders represent a significant cause of maternal morbidity and mortality worldwide.

### 4.4. Thrombotic Microangiopathies

Atypical hemolytic uremic syndrome and thrombotic thrombocytopenic purpora (TTP) are part of the thrombotic microangiopathy spectrum and are uncommon but potentially life-threatening causes of thrombocytopenia during pregnancy. These disorders are characterized by thrombocytopenia, microangiopathic hemolytic anemia, and multiorgan dysfunction resulting from extensive microvascular thrombosis [45,46,47].

The diagnosis is often challenging, as the clinical presentation may overlap with that of severe PE or HELLP syndrome. However, thrombotic microangiopathy is more frequently indicated by the presence of neurological symptoms, renal failure, and more severe thrombocytopenia [32,45,48]. Since delayed treatment beginning is linked to higher maternal mortality, early detection of these conditions is crucial. Unlike pregnancy-related conditions such as PE or HELLP syndrome, thrombotic microangiopathies require disease-specific therapies, including plasma exchange or complement inhibition, rather than delivery alone [49,50,51].

### 4.5. Other Secondary Causes

Infections, medications, induced thrombocytopenia, bone marrow issues, and systemic autoimmune diseases such as lupus erythematosus and antiphospholipid syndrome are some of the less frequent causes of thrombocytopenia during pregnancy. Viral infections such as hepatitis and human immunodeficiency virus (HIV) can cause thrombocytopenia either through immunological mechanisms or by a direct decrease in hematopoiesis [4,52,53].

The presence of additional clinical symptoms or laboratory abnormalities frequently confirms the diagnosis of thrombocytopenia. A thorough medical history, a rigorous review of drug consumption, and targeted examinations are required to identify the secondary reasons and ensure appropriate management [5].

## 5. Diagnostic Challenges and Differential Diagnosis

Diagnosing thrombocytopenia during pregnancy can be challenging due to the wide range of potential etiologies and the frequent overlap of clinical and laboratory findings. Accurate diagnosis is crucial because the underlying etiology directly influences treatment decisions and maternal and fetal outcomes [13,54]. Thus, to distinguish benign pregnant thrombocytopenia from pathological situations that necessitate immediate care, a methodical and clinically oriented approach is needed. Table 2 summarizes the key clinical and laboratory features relevant to the differential diagnosis.

The initial diagnostic evaluation should include a detailed medical history. Specifically, the investigation focused on the onset of thrombocytopenia, whether any thrombocytopenia had previously occurred, any bleeding, autoimmune conditions, medication use, and any challenges during the previous or present pregnancy [55,56]. The timing of thrombocytopenia onset is important for differential diagnosis, as autoimmune thrombocytopenia often develops before or early in pregnancy, whereas gestational thrombocytopenia typically occurs in the late second or third trimester [57]. In clinical practice, platelet count thresholds are commonly used to guide diagnostic evaluation and management decisions. Mild thrombocytopenia is generally defined as platelet counts 100–150 × 10^9^/L, moderate thrombocytopenia as 50–100 × 10^9^/L, and severe thrombocytopenia as <50 × 10^9^/L [13].

The first step in laboratory evaluation is confirmation of true thrombocytopenia. Pseudothrombocytopenia, which results from in vitro platelet aggregation—most commonly in ethylenediaminetetraacetic acid (EDTA)–anticoagulated samples—should always be excluded. Examination of a peripheral blood smear is essential to detect platelet clumping and to assess platelet morphology or the presence of schistocytes suggestive of microangiopathic hemolysis [58]. When EDTA-dependent platelet aggregation is suspected, platelet counts should be repeated using sodium citrate anticoagulated blood samples, in which platelet aggregation does not typically occur. Confirmation with citrate samples helps establish the diagnosis of pseudothrombocytopenia and prevents misdiagnosis of severe thrombocytopenia and unnecessary treatment [59].

One of the most common diagnostic challenges is distinguishing ITP from gestational thrombocytopenia, two conditions that differ significantly in management and prognosis [60]. While autoimmune thrombocytopenia may manifest as more severe thrombocytopenia, a history of the disorder, or persistently low platelet counts after delivery, gestational thrombocytopenia is defined by moderate, isolated thrombocytopenia in an otherwise healthy pregnant woman. Because antiplatelet antibody testing has low sensitivity and specificity and cannot reliably differentiate between disorders, it is generally not recommended [52].

Women presenting with thrombocytopenia in the third trimester should also be evaluated for pregnancy-related hypertensive conditions, particularly PE and HELLP syndrome, which are discussed in Section 4 [7]. On the contrary, thrombocytopenia may precede other clinical symptoms, and unusual or incomplete presentations are frequent. Thus, in cases of progressing disease, sequential laboratory monitoring is essential [5].

Another important diagnostic consideration is thrombotic microangiopathy, which may clinically resemble severe PE or HELLP syndrome but requires fundamentally different management strategies [61]. Atypical hemolytic uremic syndrome or TTP should be suspected in cases of profound thrombocytopenia, severe hemolysis, neurological symptoms, or progressive renal impairment. When clinical suspicion is high, treatment decisions should not be delayed, even though specialist tests such as ADAMTS13 activity can assist in diagnosis [62].

**Figure 1:**
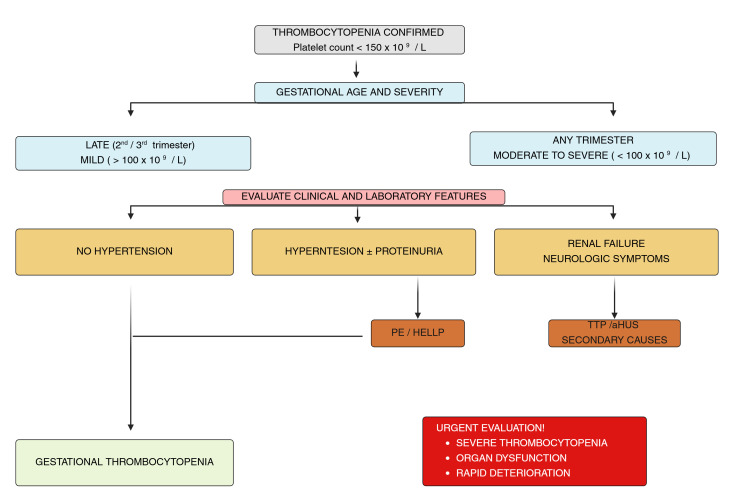
Diagnostic algorithm for thrombocytopenia in pregnancy. Flowchart illustrating the stepwise clinical evaluation of thrombocytopenia during pregnancy based on gestational age, platelet count severity, and associated clinical and laboratory findings. Abbreviations: PE, pre-eclampsia; HELLP, hemolysis, elevated liver enzymes, low platelets; TTP, thrombotic thrombocytopenic purpura; aHUS, atypical hemolytic uremic syndrome.

The diagnostic approach to thrombocytopenia in pregnancy frequently relies on a combination of clinical assessment, laboratory findings, response to initial treatment, and the subsequent postpartum course, owing to the complexity and overlap of clinical features [27]. Multidisciplinary collaboration between obstetricians, hematologists, and other specialists is essential for timely and appropriate management. A systematic diagnostic strategy contributes to accurate etiological diagnosis and to reducing the risk of incorrect or delayed therapeutic interventions and unnecessary diagnostic tests [63,64]. Table 2 and Figure 1 summarize the key elements of the differential diagnosis and the recommended diagnostic strategy.

**Table 2:** The table summarizes the main clinical and laboratory features for differentiating common causes of thrombocytopenia in pregnancy.

Condition	Typical Onset	Severity of Thrombocytopenia	Key Laboratory Findings	Distinguishing Clinical Features	Postpartum Evolution
Gestational thrombocytopenia [6,65]	Late 2nd–3rd trimester	Mild (>100 × 10^9^/L)	Isolated thrombocytopenia; normal smear	Asymptomatic; no prior history	Rapid spontaneous resolution
ITP [52,57,66]	Any trimester; often early or pre-existing	Mild to severe	Isolated thrombocytopenia; normal smear	Possible bleeding history; prior ITP	Often persists postpartum
PE [67]	Usually, 3rd trimester	Mild to moderate	Elevated liver enzymes; proteinuria	Hypertension; systemic symptoms	Improves after delivery
HELLP syndrome [7,8]	3rd trimester or postpartum	Moderate to severe	Hemolysis, ↑ AST/ALT, ↑ LDH	Epigastric pain; rapid progression	Resolves after delivery
TTP [8,68]	Late pregnancy or postpartum	Severe	Schistocytes; ↑ LDH; anemia	Neurologic symptoms; minimal hypertension	Persists without plasma exchange
aHUS [69,70]	Postpartum > antepartum	Severe	Renal failure; hemolysis	Progressive kidney injury	Often persists postpartum
Secondary causes (infection, drugs, autoimmune disease) [71]	Variable	Variable	Disease-specific abnormalities	Systemic illness or drug exposure	Depends on underlying cause

Abbreviations: HELLP, hemolysis, elevated liver enzymes, and low platelet count; LDH, lactate dehydrogenase; TTP, thrombotic thrombocytopenic purpura; aHUS, atypical hemolytic uremic syndrome; ITP, immune thrombocytopenia; AST, aspartate aminotransferase; ALT, alanine aminotransferase; ↑, increased.

## 6. Maternal Risks

The maternal risk of pregnancy-related thrombocytopenia is determined primarily by the severity of thrombocytopenia, the rate of platelet decline, and the presence of coexisting obstetric or systemic conditions. Mild thrombocytopenia is usually clinically insignificant, while moderate to severe forms are associated with increased maternal morbidity, particularly during labor and puerperium [4,67].

The most significant maternal concern is bleeding. Thrombocytopenia can increase the risk of bleeding before, during, and after delivery, particularly when the platelets count falls below the hemostatic threshold or when other risk factors, such as uterine atony, cesarean delivery, or poor blood coagulation, are present [72,73]. Thrombocytopenia may also exacerbate postpartum bleeding, potentially increasing the need for blood transfusions. Postpartum hemorrhage remains a major cause of maternal morbidity worldwide [73].

Thrombocytopenia also affects anesthetic management by limiting the use of neuraxial anesthesia [74]. In pregnant women with hypertensive disorders, airway edema, or cardiac dysfunction, thrombocytopenia may increase the risk of hemorrhagic complications during neuraxial procedures and may necessitate the use of general anesthesia, which is associated with higher maternal risk [75,76]. Consequently, thrombocytopenia may restrict anesthetic options during labor or cesarean delivery, potentially influencing maternal outcomes.

In some cases, thrombocytopenia reflects an underlying systemic disorder that, in its most severe forms, may lead to multiorgan failure. Major maternal complications include acute kidney injury, hepatic dysfunction, neurological manifestations, and disseminated intravascular coagulation. These conditions are associated with increased maternal morbidity and often require advanced medical care, including intensive care admission and prolonged hospitalization [77].

This hematological abnormality can also complicate obstetric decision-making, particularly regarding the timing and mode of delivery. The need to balance maternal safety with fetal maturity may result in preterm delivery or operative intervention. In addition, repeated laboratory monitoring, transfusion requirements, and close collaboration among multiple specialists may adversely affect both the psychological well-being and the overall health of the pregnant woman [78,79].

## 7. Fetal and Neonatal Risks

Maternal thrombocytopenia is associated with a wide range of fetal and neonatal risks, which are mainly determined by the severity of the maternal condition, gestational age at delivery, and the underlying etiology. Although thrombocytopenia during pregnancy does not affect the fetus in most cases, certain clinical conditions are associated with increased perinatal morbidity and therefore require close monitoring and coordinated perinatal care [28,80].

One of the most frequent adverse fetal outcomes linked to maternal thrombocytopenia is premature birth, particularly when early delivery is required due to maternal complications [81]. Iatrogenic preterm birth is associated with increased risks of respiratory distress, neonatal infection, and long-term neurodevelopmental complications. In addition, fetal growth restriction may occur, potentially reflecting placental dysfunction or the effects of maternal disease [82,83].

When maternal immune platelet abnormalities affect pregnancies, neonatal thrombocytopenia is a serious concern. Reduced newborn platelet counts can result from the transplacental transfer of maternal antibodies and typically appear in the first few days of life [84]. Severe thrombocytopenia necessitates close postnatal monitoring since it may raise the risk of bleeding problems, even if the majority of affected infants do not exhibit any symptoms. A rare but dangerous side effect of severe newborn thrombocytopenia is intracranial hemorrhage [85]. Avoiding needless stressful procedures during delivery is crucial because the risk is highest during the perinatal period. The evidence currently available does not support routine cesarean sections for the primary purpose of preventing newborn hemorrhage. Rather, obstetric indicators and the health of the mother should be taken into consideration while making delivery decisions [86,87].

Thrombocytopenia may also complicate obstetric decision-making, particularly regarding the timing and mode of delivery. In some cases, early delivery may be required to prevent maternal deterioration while balancing the goal of achieving adequate fetal maturity. The need for transfusions, frequent checkups, and collaboration between different specialists cannot only influence the psychological state of the pregnant woman but also affect her overall health [31,67,88].

Overall, optimal outcomes depend on timely diagnosis, multidisciplinary collaboration, and careful perinatal monitoring, particularly in pregnancies complicated by severe maternal disease [28,31,56].

## 8. Management Strategies

Pregnancy-related thrombocytopenia management necessitates a customized strategy based on the underlying cause, thrombocytopenia severity, gestational age, and maternal and fetal condition [89,90]. The primary goals of management are to prevent unnecessary interventions, safeguard the fetus, and lower the mother’s risk of bleeding while facilitating a safe delivery. An interdisciplinary team of neonatologists, obstetricians, hematologists, and anesthesiologists should make management decisions because of the range of etiologies and clinical manifestations [91]. Table 3 presents a summary of management techniques by etiology.

### 8.1. General Management Principles

Close clinical and laboratory monitoring is crucial for all pregnant women with thrombocytopenia. Serial platelet counts provide valuable insights into the progression of the condition and the effectiveness of treatment [52,94]. While severe or progressive thrombocytopenia requires targeted management, monitoring alone is often adequate for asymptomatic individuals with mild thrombocytopenia [95].

The therapeutic approach to thrombocytopenia during pregnancy should be guided by the overall clinical context, including bleeding symptoms, platelet count, and planned obstetric interventions, rather than relying solely on laboratory parameters [66]. In the absence of bleeding, prophylactic therapy is generally not recommended unless the platelet counts fall below safe thresholds for delivery or invasive procedures. Avoiding unnecessary platelet transfusions is particularly important due to their limited duration of effect and potential complications [96,97].

### 8.2. Etiology-Specific Management

Depending on the underlying etiology, different management approaches are used. In cases of gestational thrombocytopenia, no specific therapy is required beyond routine monitoring [98]. Patients with symptomatic bleeding or platelet counts below safe limits are advised to receive treatment for immune-mediated thrombocytopenia, especially at the end of pregnancy when delivery is anticipated. The goal of first-line treatments is to quickly raise platelet levels for safe labor management [99,100].

The main treatment for thrombocytopenia linked to pregnancy-related hypertension diseases is to stabilize the mother’s state and, if necessary, give birth early. The core of management is supportive care, which includes controlling blood pressure and correcting coagulation abnormalities [101,102]. On the other hand, thrombotic microangiopathy should be treated in specialized facilities and calls for immediate, targeted care. Delaying treatment can lead to unfavorable outcomes and rapid clinical deterioration [103,104].

Management of immune-mediated thrombocytopenia during pregnancy depends on disease severity, platelet count, gestational age, and the presence of bleeding symptoms. Treatment is generally recommended in cases of symptomatic bleeding or when platelet counts fall below safe thresholds for delivery or invasive procedures [57,66]. First-line therapy includes corticosteroids and IVIG, both of which are considered relatively safe during pregnancy [66,103]. Corticosteroids are frequently used during the first and second trimesters but require careful monitoring due to potential maternal adverse effects such as gestational diabetes or hypertension. IVIG is often preferred when a rapid increase in platelet count is required or when corticosteroids are contraindicated [57,66,103].

In refractory cases, second-line treatments such as rituximab or thrombopoietin receptor agonists may be considered in specialized centers after careful evaluation of maternal and fetal risks [103]. Management decisions should always be individualized and made within a multidisciplinary team [66].

Secondary causes of thrombocytopenia require treatment of the underlying condition, including management of systemic autoimmune diseases, discontinuation of causative medications, or treatment of infections. Platelet counts typically recover once the triggering factor has been addressed [105].

### 8.3. Platelet Transfusion and Supportive Therapies

Platelet transfusions are generally reserved for patients with severe thrombocytopenia, active bleeding, or those requiring invasive procedures or emergency delivery [106]. Platelet transfusion is most effective when used as an adjunct to definitive treatment of the underlying disorder, providing a temporary increase in platelet count. Decisions regarding transfusion thresholds should consider the overall clinical context, procedural requirements, and risk of bleeding [96,107].

In ITP, platelet transfusion alone may be less effective because transfused platelets can be rapidly destroyed by circulating antiplatelet antibodies. Therefore, platelet transfusions are typically administered in combination with immunomodulatory therapy, such as corticosteroids or IVIG, particularly in cases of severe bleeding or when urgent delivery or invasive procedures are required [66,103]. This combined approach may improve platelet survival and reduce bleeding risk.

Supportive care includes iron supplementation, red blood cell transfusions for anemia, and treatment of hemostatic disorders when necessary. These interventions aim to reduce bleeding complications during delivery and optimize maternal hemostasis [108,109].

### 8.4. Delivery Planning and Peripartum Management

Pregnancies complicated by thrombocytopenia require careful planning is necessary of delivery under multidisciplinary supervision. Decisions regarding the timing and mode of delivery should consider not only on the platelet count but also the patient’s clinical condition and obstetric indications. When obstetrically appropriate, vaginal delivery is generally preferred, as it is associated with a lower risk of surgical bleeding compared with cesarean delivery [67,110].

Effective multidisciplinary collaboration is essential in the peripartum management of these pregnancies, ensuring appropriate monitoring, availability of blood products, and preparedness for neonatal care. Postnatal follow-up of the neonate is necessary to assess platelet recovery, reassess the diagnosis, and provide counseling regarding the risk of recurrence in subsequent pregnancies [111].

## 9. Delivery and Anesthetic Considerations

To balance procedural risks, fetal well-being, and maternal safety, careful multidisciplinary coordination is necessary for the management of labor in pregnancies complicated by thrombocytopenia. Decisions regarding the timing and mode of delivery, as well as the choice of anesthesia, should be individualized and guided by platelet count trends, obstetric indications, and the overall clinical context [112,113].

### 9.1. Mode and Timing of Delivery

Thrombocytopenia alone is not an indication for cesarean delivery. When obstetrically appropriate, vaginal delivery is generally preferred because it is associated with a lower risk of surgical complications and maternal blood loss [114]. To reduce trauma-related bleeding, especially cerebral hemorrhage, surgical vaginal delivery may be avoided in cases of severe maternal or expected newborn thrombocytopenia [115].

The timing of delivery should take into account both maternal stability and fetal maturity. In some cases, early delivery may be required to prevent maternal clinic deterioration, whereas in other situations expectant management with close monitoring may allow further fetal maturation. Careful delivery planning also facilitates multidisciplinary preparation, including the availability of blood products and neonatal support [14,116,117].

### 9.2. Platelet Thresholds for Delivery

Although universally accepted platelet thresholds are lacking, several clinical thresholds are commonly used to guide peripartum management [118]. In general, lower platelet counts may be acceptable for vaginal delivery, whereas higher platelet levels are often preferred for cesarean delivery or other invasive procedures in order to reduce the risk of surgical bleeding. When interpreting platelet thresholds, clinicians should also consider coexisting coagulation abnormalities, bleeding history, platelet function, and the underlying cause of thrombocytopenia [119,120].

### 9.3. Neuraxial and General Anesthesia Considerations

A neuraxial anesthesia risk assessment in thrombocytopenic patients should be meticulous, considering the risk of a spinal or epidural hematoma. Anesthetic choice should reflect the stability of platelet counts, the cause of thrombocytopenia, and the absence of additional bleeding risk factors. If neuraxial anesthesia is not an option, general anesthesia might be necessary, with increased vigilance for hemodynamic, airway, and respiratory issues, especially in the obstetric environment [121,122].

Clear and continuous communication between obstetric and anesthesiology teams is essential for perinatal safety, facilitating prompt decision-making and reducing the risk of adverse events during labor. If possible, scheduled deliveries are beneficial in reducing emergency anesthetic situations and in allowing for platelet count optimization [123,124].

### 9.4. Neonatal Considerations at Delivery

Early notification of the neonatal team is crucial when maternal thrombocytopenia may impact neonatal care. Neonates are considered at increased risk particularly when maternal thrombocytopenia is associated with immune-mediated conditions, such as ITP, or when severe maternal thrombocytopenia is present near delivery (e.g., platelet count < 50 × 10^9^/L). In these cases, neonates should be closely monitored in the early postnatal period, and cord blood platelet counts may be obtained selectively. Although obstetric practice aims to minimize fetal stress, current evidence does not support routine modification of the mode of delivery to prevent neonatal thrombocytopenia [80].

Current consensus statements suggest that neuraxial anesthesia can generally be considered safe in obstetric patients with stable platelet counts of ≥70 × 10^9^/L, provided that no additional coagulopathy or bleeding risk factors are present. Decisions should always be individualized and made in collaboration with anesthesiology teams, taking into account platelet trends, clinical context, and the underlying cause of thrombocytopenia.

## 10. Postpartum Management and Follow-Up

The postpartum period is an important time for the evaluation and management of pregnancy-related thrombocytopenia, as changes in platelet counts after delivery may provide valuable diagnostic and prognostic information. Whether platelet counts return to normal or remain low after delivery can help clarify the underlying etiology and determine whether further monitoring or treatment is required [30].

To assess the course of thrombocytopenia, platelet counts should be reassessed shortly after delivery and again during the routine postnatal follow-up visit [73]. Gestational thrombocytopenia usually resolves spontaneously within a few weeks after delivery. However, persistent or worsening thrombocytopenia remains may suggest an immune-mediated or systemic disorder and should prompt further diagnostic evaluation. Women who experienced severe thrombocytopenia or required treatment during pregnancy should therefore receive close postpartum monitoring [6,10].

During early postpartum period, women with thrombocytopenia require careful clinical observation because of the increased risk of postpartum hemorrhage. Systematic assessment of uterine tone, blood loss, and hemodynamic stability is particularly important when additional risk factors are present [119].

Clinical presentation and laboratory data, rather than just predetermined thresholds, should be used to inform decisions on the delivery of blood products. Furthermore, long-term hematological management may be necessary for persistent or recurrent thrombocytopenia, and modifying or stopping pregnancy-related medicine is a crucial component of postpartum care [113,125].

An important aspect of postpartum care is counseling women about the potential risk of recurrence in future pregnancies [126]. Certain forms of thrombocytopenia may recur in subsequent pregnancies, although the clinical presentation and severity may vary. Early antenatal assessment and appropriate monitoring are therefore recommended for women with a history of severe thrombocytopenia [127].

In addition to maternal follow-up, neonatal outcomes should also be monitored when maternal thrombocytopenia is related to immune-mediated conditions. Neonates born to mothers with ITP may develop transient thrombocytopenia during the first days of life due to transplacental transfer of antiplatelet antibodies, and therefore early platelet count assessment and clinical monitoring are recommended [41,82]. Although most cases resolve spontaneously, follow-up may be required in severe cases to ensure recovery of platelet counts and to prevent bleeding complications [86,87]. Counseling regarding the risk of recurrence in future pregnancies, particularly in cases of ITP or pregnancy-associated thrombotic microangiopathies, is also an important component of postpartum care [37,65].

## 11. Future Directions and Research Gaps

While many questions remain unanswered, significant advancements have been made in understanding and treating thrombocytopenia during pregnancy [128]. Due to the ethical and practical constraints of conducting randomized controlled trials in pregnant women, the current evidence base mainly consists of observational studies, retrospective analyses, and expert consensus [129]. Consequently, individualized risk assessment remains a key component of many therapeutic decisions.

An area that has received considerable attention is the definition of safe platelet thresholds for neuraxial anesthesia and obstetric procedures, as current guidelines differ widely. To provide more precise, evidence-based recommendations, prospective studies are needed to examine maternal, anesthetic, and perinatal outcomes at different platelet levels [130,131].

Despite growing clinical experience, several aspects of thrombocytopenia management in pregnancy remain controversial. One example is the optimal platelet threshold for neuraxial anesthesia, where recommendations differ among professional societies and are often based on observational data rather than randomized trials [76,117,125]. Similarly, uncertainty remains regarding the most appropriate treatment thresholds for ITP during pregnancy and the safety of emerging therapeutic agents. These variations reflect the limited availability of high-quality prospective studies in pregnant populations and highlight the need for more robust evidence to guide clinical decision-making [131].

The assessment of new treatment options for expectant mothers is equally important. Although patients with thrombocytopenia who are not pregnant now have many more therapy options thanks to newer immunomodulatory and targeted drugs, less is known regarding their safety, efficacy, and potential long-term effects on the developing fetus [132,133,134]. It is crucial to perform long-term monitoring of newborns who have been exposed and to systematically collect safety data specific to pregnancy to support evidence-based clinical practice moving forward.

Another important field of research appears to be the development of improved diagnostic instruments. The use of biomarkers that can clearly distinguish between conditions with overlapping clinical features, such as thrombotic microangiopathy and hypertensive disorders of pregnancy, may therefore expedite diagnosis and guide treatment more effectively [32]. Additionally, a major step toward improving outcomes and reducing clinical care variation may be the adoption of uniform diagnostic procedures that work for a variety of groups.

There is a notable gap in the literature concerning patient-centered outcomes, including psychological burden, quality of life, and preferences in therapeutic decision-making. Future research that incorporates these measures may facilitate shared decision-making and support a more comprehensive, patient-oriented approach to managing thrombocytopenia in pregnancy [135,136].

## 12. Conclusions

Pregnancy-related thrombocytopenia is a common observation, although it can be caused by several disorders, ranging from normal pregnancy to serious conditions that could jeopardize the mother’s and the fetus’s health. Therefore, identifying the underlying cause is essential, as treatment and prognosis vary significantly among different conditions. In particular, such conditions that are linked to a markedly elevated risk of maternal and fetal morbidity and mortality necessitate extremely keen awareness and meticulous clinical evaluation.

This research focuses on the efficient management of thrombocytopenic pregnant women through a stepwise diagnostic workup, customized medication, and close coordination among various experts. Ultimately, thorough consideration of fetal and newborn health, careful assessment of maternal risks, and suitable delivery planning will yield the best results.

Progress in addressing thrombocytopenia during pregnancy requires robust pregnancy-specific diagnostic and therapeutic evidence, supported by high-quality, evidence-based clinical guidelines. To provide the greatest potential maternal and perinatal outcomes, the best care will likely be provided in the interim by judiciously combining fresh evidence, established recommendations, and individualized clinical judgment.

## Figures and Tables

**Table 1:** Major causes of thrombocytopenia in pregnancy and their key clinical characteristics.

Etiology	Prevalence	Typical Timing	Severity	Key Features	Postpartum Course
Gestational thrombocytopenia [30]	70–80%	Late 2nd–3rd trimester	Mild (usually >100 × 10^9^/L)	Hemodilution, increased placental consumption, enhanced platelet clearance	Spontaneous resolution within weeks
ITP [30,31]	3–5%	Any trimester; often early or pre-existing	Mild to severe	Autoantibody-mediated platelet destruction and impaired production	May persist postpartum
Hypertensive disorders (e.g., PE, HELLP syndrome) [30,32]	~5–10%	Usually 3rd trimester	Mild to severe (HELLP often severe)	Endothelial dysfunction, platelet activation, and consumption	Improves after delivery
Thrombotic microangiopathies (TTP, aHUS) [33]	Rare	Late pregnancy or postpartum	Severe	Microvascular thrombosis, hemolysis, organ injury	Often persists postpartum
Other secondary causes (infection, drugs, autoimmune disease, marrow disorders) [3,34]	Uncommon	Variable	Variable	Immune-mediated destruction, marrow suppression, or systemic disease	Depends on etiology

Abbreviations: HELLP, hemolysis, elevated liver enzymes, and low platelet count; TTP, TTPmbocytopenic purpura; aHUS, atypical hemolytic uremic syndrome.

**Table 3:** The table summarizes general management principles according to etiology, including indications for treatment, use of platelet transfusion, and key peripartum considerations. Management should be individualized based on disease severity, gestational age, and maternal–fetal status.

Etiology	Primary Management Approach	Indications for Treatment	Role of Platelet Transfusion	Key Peripartum Considerations
Gestational thrombocytopenia [4,6]	Observation and routine monitoring	None	Not indicated	Vaginal delivery preferred; no special interventions required
ITP [38,57]	Immunomodulatory therapy when indicated	Symptomatic bleeding or platelet count below safe thresholds for delivery/procedures	Reserved for active bleeding or urgent delivery	Aim to optimize platelet count near delivery; neonatal monitoring required
Hypertensive disorders (PE, HELLP syndrome) [5,92]	Maternal stabilization and timely delivery	Progressive disease or maternal/fetal compromise	Used selectively in severe thrombocytopenia or bleeding	Delivery is definitive treatment; anticipate postpartum recovery
Thrombotic microangiopathies (TTP, aHUS) [32,47]	Urgent disease-specific therapy in specialized centers	Immediate upon clinical suspicion	Adjunctive only; limited efficacy alone	Delivery alone insufficient; intensive monitoring required
Secondary causes (infection, drugs, autoimmune disease) [52,93]	Treatment of underlying condition	Based on disease severity	Case-dependent	Platelet recovery parallels resolution of cause

Abbreviations: HELLP, hemolysis, elevated liver enzymes, and low platelet count; TTP, thrombotic thrombocytopenic purpura; aHUS, atypical hemolytic uremic syndrome.

## Data Availability

No new data were created or analyzed in this study.

## Abbreviations

The following abbreviations are used in this manuscript:

HELLP	Hemolysis, Elevated Liver enzymes & Low Platelets
PE	Preeclampsia
ITP	Immune thrombocytopenia
aHUS	Atypical hemolytic uremic syndrome
TTP	Thrombotic thrombocytopenic purpura
HIV	Human immunodeficiency virus
LDH	Lactate dehydrogenase
LFTs	Liver function tests
